# Risk factors associated to asthma in 6-7 year old schoolchildren of Rio de Janeiro, Brazil

**DOI:** 10.1186/1939-4551-8-S1-A160

**Published:** 2015-04-08

**Authors:** Solange Oliveira Rodrigues Valle, Fabio Kuschnir, Dirceu Sole, Antonio Ledo Alves Cunha

**Affiliations:** 1 Hospital Universitário Clementino Fraga Filho Hucff-Ufrj, Brazil; 2 Rio De Janeiro State University, Brazil; 3 Brazilian Society, Brazil; 4 Federal University of Rio De Janeiro, Rio De Janeiro, Brazil

## Background

Asthma has a high prevalence in Brazil, however there are few reports on the factors associated with this disease in our country. The aim of this study was to evaluate the risk factors for asthma in children aged 6-7 years old from the city of Rio de Janeiro

## Methods

Cross-sectional study using the adapted and validated ISAAC Written Questionnaire for Asthma for telephone interview. The sample was the systematic random type without replacement for school classes and students. Data were collected by a polling company from May/July 2010. Bivariate analyses between asthma ("wheezing in the last 12 months") and the study factors were performed using odds-ratio (OR), confidence intervals of 95% (95%CI) and Chi-square test. Factors associated to asthma in a bivariate analysis were studied using logistic regression models. The adopted level of significance was 5%.

## Results

3,216 interviews (51.4% boys) were analyzed. Mothers were the main respondents (71.9%). The prevalence of asthma was 20.9%. Male sex (OR=1.37; 95%CI:1.14-1.64);presence of rhinitis (OR=3.12; 95%CI:2.59-3.76);report of worms (OR=1.28; 95%CI:1.04-1.57); Maternal asthma and rhinitis(OR=1.63; 95%CI:1.15-2.21 and OR=1.30;95%CI:1.03-1.65), exposure to maternal smoking during the first year of life and current (OR=1.43;95%CI:1.12-1.81 and OR=1.37;95%CI:1.10-1.72) and both the presence of mold in domicile during the first year of life and current (OR=1.63;95%CI:1.35-1.97 and OR=1.35; 95%CI:1.11-1.64) were independently associated to asthma.

## Conclusions

genetic and environmental factors were associated to asthma in children from Rio de Janeiro city. Early Interventions on these last factors may decrease the occurrence of asthma in this population.

